# Evaluating the Mentzer Index for Screening of Iron Deficiency Anemia and Beta Thalassemia Among Infants Visiting Primary Health Centers in Dubai, United Arab Emirates: A Retrospective Study

**DOI:** 10.7759/cureus.66286

**Published:** 2024-08-06

**Authors:** Alya Alkamali, Latifa S Alshafiei, Maryam AlJasmi, Hadi Helali, Idris Alhmid, Fatima AlOlama, Fatima Mazahir

**Affiliations:** 1 Pediatrics, Mohammed Bin Rashid University of Medicine and Health Sciences, Dubai, ARE; 2 Pediatrics, Al Jalila Children's Speciality Hospital, Dubai, ARE; 3 Pediatrics, Dubai Health Authority, Dubai, ARE; 4 Family Medicine, Dubai Health Authority, Dubai, ARE

**Keywords:** anemia, primary healthcare centers, screening program, mentzer index, β-thalassemia, iron deficiency anemia (ida)

## Abstract

Objective: The study aims to apply the Mentzer index to the population of all eligible pediatric patients presenting to primary healthcare centers (PHCs) in Dubai for the first year of life screening. Additionally, the study will estimate the prevalence of iron deficiency anemia (IDA) and β-thalassemia in children presenting to the PHCs and evaluate the importance of PHCs in screening children for IDA and β-thalassemia by comparing the results of this study to previous results.

Methods: The SALAMA system (electronic medical record system used in PHCs in Dubai) was used for collecting the data. Eligible patients’ data, such as hemoglobin, mean corpuscular volume (MCV), and red blood cell (RBC) counts were collected and recorded. The Mentzer index was applied to patients and the index results were compared to the gold standard results extracted from the SALAMA system. The gold standard diagnosis for IDA used was the serum ferritin test and the gold standard diagnosis for β-thalassemia used in the study was hemoglobin electrophoresis.

Results: Out of the 75 eligible patients with low hemoglobin, 11 (14.6%) had low ferritin indicating IDA. Moreover, eight (10.6%) patients had abnormal hemoglobin electrophoresis indicating β-thalassemia. The Mentzer index was applied to the patients; 13 (17.3%) of them had a value less than 13 (β-thalassemia) and 6 (8%) had a value more than 13 (IDA). The sensitivity of the Menzter index in screening IDA and β-thalassemia is 99% and the specificity is 54.5%.

Conclusion: Our study found that the Mentzer's index is a reliable screening tool due to its high sensitivity; however, we suggest replicating the study with a larger sample size to get more clinically significant results.

## Introduction

Studies have shown a high prevalence of iron deficiency anemia (IDA) and β-thalassemia in the Middle East, making them relevant diseases [1]. Nearly all Arab nations see polymorphic occurrences of β-thalassemia, with carrier rates ranging from 1% to 11% [2]. The prevalence of β-thalassemia in the United Arab Emirates (UAE) is 8.5% which is a serious health issue given that the majority of β-thalassemia mutations in the UAE are extremely severe [3]. The prevalence of iron deficiency is higher in infants in Arab countries at about 72%. Similarly, the incidence rate of IDA in infants in the UAE was reported to be about 23.2% [4].

IDA is defined as a microcytic (mean corpuscular volume (MCV) is less than 80 fL), hypochromic (mean corpuscular hemoglobin (MCH) is less than 27 pg) anemia [5]. The elected initial diagnostic test for IDA is the serum ferritin test [6]. β-thalassemia is also considered a microcytic, hypochromic anemia [7]. It is an autosomal recessive disorder characterized by a defect in the synthesis of the beta (β) globin chain in hemoglobin [8]. The preferred method of diagnosis for β-thalassemia is a complete blood count (CBC) followed by a hemoglobin electrophoresis test [4].

In the first year of life, infants are required to undergo screening for certain harmful or potentially fatal disorders that aren’t otherwise apparent at birth [9]. The screening investigates the presence of blood disorders which include IDA and β-thalassemia [9]. The process involves performing a CBC [10]. If any abnormalities are detected, additional tests are ordered to further investigate. The standard process is very lengthy and demanding [11]. Alternative indices could be used for the diagnosis and differentiation of IDA and β-thalassemia [12]; however, they are not normally used in standard practice due to insufficient research or non-superiority when compared to the gold standard tests for both IDA and β-thalassemia [13]. The study will be performed to test the reliability and validity of the Mentzer index in screening IDA and β-thalassemia in primary healthcare centers (PHCs) in Dubai, UAE among infants.

The objective of the study is to apply the Mentzer index to the population of all eligible pediatric patients presenting to PHCs in Dubai for the first year of life screening. Additionally, the study will estimate the prevalence of IDA and β-thalassemia in children presenting to the PHCs and evaluate the importance of PHCs in screening children for IDA and β-thalassemia. There was only a single study performed evaluating the significance of the Menzter index in detecting IDA in Hatta Hospital, Hatta, UAE; however, the results of that study could not be generalized to a large population since a bigger sample size was needed. Additionally, the study done in Hatta Hospital did not compare the accuracy of the Mentzer index in screening both IDA and β-thalassemia since the study only assessed IDA [14]. To be able to apply the Mentzer index to practice in the UAE, a retrospective citywide or even nationwide study was needed to obtain a sufficient sample size.

## Materials and methods

Study design

Retrospective cohort data analysis was performed on pediatric patients presenting to PHCs with abnormalities in their CBC. All eligible pediatric patients presenting to the PHCs between January 2019 to December 2022 were included.

Study setting

Data from 13 PHCs regarding pediatric patients aged 11 to 18 months coming in for their first year of life screening was obtained from the Electronic Medical Record System (SALAMA) used by the Dubai Health Authority (DHA).

Study size and participants

Patients between the ages of 11 to 18 months, with low hemoglobin levels, who visited PHCs between January 1, 2019, and December 31, 2022, were included in the study. Exclusions were filtered out.

Eligibility criteria

The study included male and female infants with no known genetic diseases or syndromes (Down syndrome, cystic fibrosis, sickle cell anemia, etc.). The infants were both breastfed or formula-fed, and the age of the infants was between 11 to 18 months of age.

The study excluded infants with known genetic diseases or syndromes (Down syndrome, cystic fibrosis, sickle cell anemia, etc.). Patients younger than 11 months and older than 18 months of age were filtered out. Patients outside of Dubai and in any private healthcare facility were also excluded. The study excluded all pediatric patients from the age group of interest who visited any tertiary hospital, as the research only looked at data collected from PHCs.

Data sources/measurement

In this research, data collection was conducted through access to the SALAMA system. Patients were initially filtered using the Slicer Dicer tool to include those who are currently alive and have undergone CBC tests between January 1, 2019, and December 31, 2022. Subsequently, each patient's medical records were thoroughly reviewed to identify patients who had undergone the first year of life screening between the ages of 11 to 18 months. Patients with low hemoglobin levels were detected, and documented in an Excel spreadsheet, and their MCV and red blood cell count (RBC) were recorded. Additionally, relevant patient information was gathered which included gestational age, gender, nationality, whether they were breastfed or formula-fed, and the age at which tests were conducted.

Following data collection, hemoglobin electrophoresis results and serum ferritin levels were collected from eligible patients. The patients were then filtered based on low serum ferritin levels and abnormal hemoglobin electrophoresis results. Upon completion of data collection, all needed data was exported to the IBM SPSS Statistics for Windows, Version 28 (Released 2021; IBM Corp., Armonk, New York, United States), and variables such as gender, nationality, hemoglobin, ferritin, MCV, RBC, and gestational age were coded appropriately. To address the missing gestational age data, regression analysis was employed using hemoglobin which has a strong correlation with gestational age for prediction. Statistical analyses, including the calculation of mean gestational age and percentages of genders and nationalities were performed. The Mentzer index, an equation that divides MCV by RBC, was applied using SPSS, and patients were classified based on the results; less than 13 indicating β-thalassemia, and more than 13 indicating IDA. Mentzer index results were compared with the gold standard tests used to diagnose IDA (low serum ferritin) and β-thalassemia (abnormal hemoglobin electrophoresis) using contingency tables. Finally, sensitivity, specificity, and confidence intervals (CI) were calculated [15].

This study was reviewed by the Institutional Review Board (IRB) of Mohammed Bin Rashid University of Medicine and Health Sciences (MBRU) Student Research Projects (SRP) committee (MBRU-IRB-SRP-20-2023). This study follows the STARD reporting protocol.

## Results

Participants

**Figure 1:**
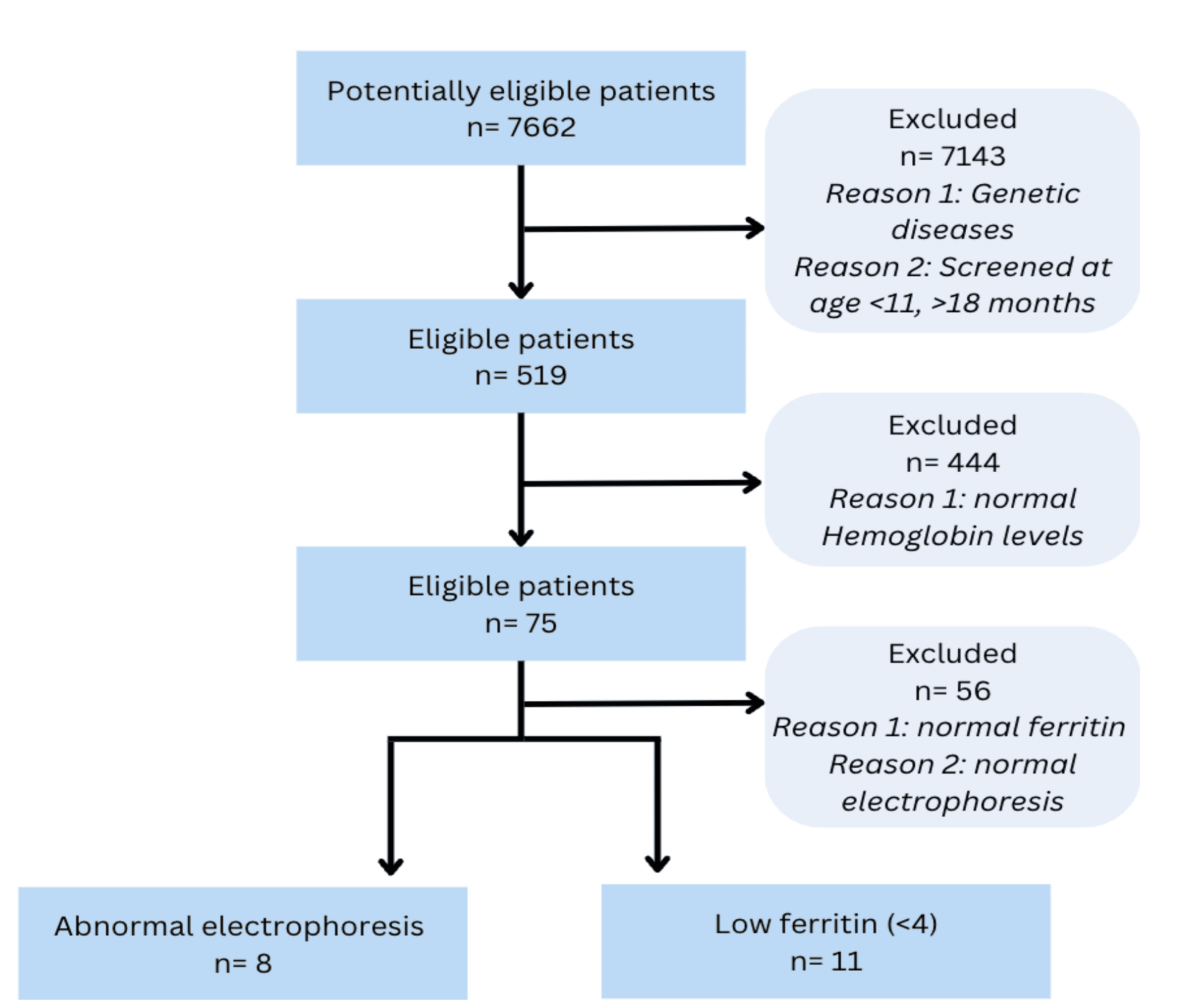
Sample size obtained flow chart based on inclusion and exclusion criteria

In the SALAMA system, a total of 7662 patients underwent CBC assessments between January 1, 2019, and December 31, 2022. Out of these, 7143 individuals were excluded due to the presence of genetic diseases or having undergone first-year-of-life screening either prior to 11 months of age or after 18 months of age. Among the remaining 519 patients, 75 had low hemoglobin levels, leading to the exclusion of the remaining cases. Of the 75, 11 patients had IDA, while 8 individuals were diagnosed with β-thalassemia based on the gold standard test results, serum ferritin, and hemoglobin electrophoresis for IDA and β-thalassemia, respectively (Figure 1).

Descriptive data

Seventy-five children were enrolled in the study. Their mean age was 14 months and 2 days, ranging from 11 months to 18 months. Only 39 (52%) children were UAE nationals, the rest were expatriates representing various ethnicities all over the world. Twenty-five (33.3%) patients were females and 50 (66.7%) were males.

Outcome data

The Mentzer index was applied to all 75 patients with low hemoglobin. Table 1 compares the serum ferritin results of the 75 patients to the Mentzer index results. The table shows that 11 (14.6%) patients had low hemoglobin with low serum ferritin (IDA) while the rest (64) had low hemoglobin with normal serum ferritin values. Out of the 11 patients with IDA, Mentzer diagnosed six with IDA (true positive) and the other five with β-thalassemia (false negative). Additionally, 40 patients with normal serum ferritin were incorrectly classified as having IDA by the Mentzer index. The prevalence of IDA is 14.6% (CI: 6.6%-22.6%).

**Table 1:** The Mentzer index diagnosis compared to the serum ferritin results IDA: iron deficiency anemia; β-thalassemia: beta thalassemia

	Low ferritin (<4 ng/mL)	Normal ferritin (>4 ng/mL)	Total
Mentzer: >13 (IDA)	6	40	46
Mentzer: <13 (β-thalassemia)	5	24	29
Total	11	64	75

Table 2 compares the hemoglobin electrophoresis results of 75 patients to Mentzer index results. The table shows that eight (10.6%) patients have β-thalassemia according to the gold standard (abnormal hemoglobin electrophoresis results). All eight were classified as having β-thalassemia by the Mentzer index (true positive). Additionally, the Mentzer index incorrectly classified 21 patients who had normal hemoglobin electrophoresis results with β-thalassemia. The prevalence of β-thalassemia is 10.6% (CI: 3.6%-17.6%).

**Table 2:** The Mentzer index diagnosis compared to the hemoglobin electrophoresis results IDA: iron deficiency anemia; β-thalassemia: beta thalassemia

	Abnormal electrophoresis	Normal electrophoresis	Total
Mentzer: β-thalassemia	8	21	29
Mentzer: IDA	0	46	46
Total	8	67	75

Table 3 excluded all anemic patients who don’t have IDA or β-thalassemia. There are a total of 19 patients with either IDA or β-thalassemia. They grouped as follows: 13 (68.4%) children were diagnosed with β-thalassemia by the Mentzer index, and six (31.5%) children were diagnosed with IDA by the Mentzer index. The index correctly diagnosed eight out of eight (100%) patients with β-thalassemia (true positive); it also correctly diagnosed 6 out of 11 (54.5%) patients who have IDA (true negative). The Mentzer Index misclassified five (45.4%) children who tested positive for IDA (low serum ferritin) as having β-thalassemia.

**Table 3:** Patients diagnosed with either IDA or β-thalassemia by the gold standard compared to the Mentzer index diagnosis IDA: iron deficiency anemia; β-thalassemia: beta thalassemia; TP: true positive; FP: false positive; FN: false negative; FN: false negative

	Gold standard: β-thalassemia	Gold standard: IDA	Total
Mentzer: β-thalassemia	8 (42.2%) (TP)	5 (26.2%) (FP)	13 (68.4%)
Mentzer: IDA	0 (0%) (FN)	6 (31.5%) (TN)	6 (31.6%)
Total	8 (42.2%)	11 (57.8%)	19 (100%)

The sensitivity of the Mentzer index in identifying IDA and β-thalassemia was 99% (CI: 94.5%-99%). The specificity of the Mentzer index in identifying IDA and β-thalassemia was 54.5% (CI: 25%-84%). Mentzer index had a positive predictive value (PPV) of 61.54% (CI: 45.58%-75.35%) and a negative predictive value (NPV) of 99% (CI: 54%-99%) in correctly diagnosing IDA and β-thalassemia.

## Discussion

Summary of major findings

The Mentzer index is used to diagnose and differentiate between IDA and β-thalassemia. If the results come out as less than 13, then the patient would be diagnosed with β-thalassemia as per the Mentzer index. If the results are more than 13, then the patient would be diagnosed with IDA.

The Mentzer index classifies patients with IDA or β-thalassemia. Since the Mentzer index does not account for any other types of anemia, a significant number of patients with neither IDA nor β-thalassemia were misdiagnosed. The Mentzer index should incorporate a category for patients who do not have IDA or β-thalassemia. Additionally, the Mentzer index does not account for patients with both IDA and β-thalassemia.

Five out of the eleven patients with IDA were misdiagnosed as having β-thalassemia. The other 6 out of 11 were correctly diagnosed as having IDA by the Mentzer index. While eight out of eight patients with β-thalassemia were accurately diagnosed by the Mentzer index, this proves that the Mentzer index is beneficial in diagnosing β-thalassemia, but not IDA.

The Mentzer index has a sensitivity of 99% (CI: 94.5%-99%) and specificity of 54.5% (CI: 24%-84%) in diagnosing and differentiating between IDA and β-thalassemia. The Mentzer index proved to be moderately reliable, giving us a CI of 25%-84% bearing in mind the small sample size; however, we believe that if the study was replicated on a larger sample size (potentially across the UAE) it would provide a narrower CI; better results in terms of validity and reliability [16]. If the study was applied with a wider inclusion criteria and involved a greater number of centers then it will give us a better indication of sensitivity and specificity; therefore, revealing exactly if the Mentzer index can play an integral role in PHCs. A better future study would be a randomized control trial testing the Mentzer index on a much larger population such as the whole Emirate of Dubai or the UAE.

The first year of life screening investigates the presence of blood and genetic disorders which include IDA and β-thalassemia. The process involves performing a CBC which starts by obtaining a blood sample from the infant. If any abnormalities are detected, such as a low hemoglobin value, a serum ferritin test will be ordered to further investigate whether the low hemoglobin level can be attributable to IDA, this involves taking another blood sample from the infant. If serum ferritin levels appear to be normal, a hemoglobin electrophoresis test will be ordered by the pediatrician to further assess if the patient has β-thalassemia. The hemoglobin electrophoresis would also require another blood sample indicating that the child has to be pricked again. This proves that the standard process is both very lengthy and invasive for the child. By utilizing the Mentzer index during the first year of life screening at PHCs, there is a notable reduction in the amount of time and resources needed to reach the final diagnosis.

The Mentzer index can be used as a substitute for the standard demanding and time-consuming procedures currently implemented at PHCs. If implemented in PHCs, the Mentzer index can be used as a screening tool due to its high sensitivity.

Ideally, we would want to see an additional function added to the SALAMA system labeled Mentzer index that would allow for automatic calculation of the Mentzer index for the attending physician upon receipt of MCV and RBC from the CBC. The main strength of our study is to provide an alternative diagnostic method that can assist the CBC performed at the first year of life screening to further guide the diagnosis of IDA and β-thalassemia. Additionally, we are evaluating the Mentzer index’s ability to replace the current standard tests that have been proven to be invasive and time-consuming; furthermore, by eliminating these barriers we will be reaching early and accurate diagnosis which could annihilate the limitation of attrition. Investigations are required in the first year of life due to the remarkable rise in the number of cases. The prevalence of β-thalassemia in the UAE is 8.5%. Similarly, the incidence rate of IDA in infants in the UAE was reported to be about 23.2% [4]. It is crucial to minimize the limitations that come alongside the standard tests, while still maintaining the level of reliability and validity of the diagnostic tools used; therefore, the Mentzer index was proven to be a valuable alternative diagnostic tool when it comes to diagnosing β-thalassemia and IDA.

Comparison with previous studies

The prevalence of IDA in our study is 14.6% and the prevalence of β-thalassemia is 10.6% out of 75 anemic patients. The sample size used in this study is not an accurate representation of Dubai’s population or the UAE as a whole due to the insufficient number of patients. This study solely targeted children who had abnormal CBC results from their first year of life screening at PHCs. To be able to accurately test the validity of the Mentzer index, a retrospective study on a wider scale should be performed. In the UAE, there is one other study that used the Mentzer index to screen children for IDA in Hatta; however, the results of that study could not be generalized as they had a small sample size confined to Hatta. A bigger sample size is required to obtain sufficient information. Additionally, the study only assessed IDA, it ceased to compare the accuracy of the Mentzer index in diagnosing both IDA and β-thalassemia. In the study conducted in Hatta, the prevalence of IDA was 23% of 99 anemic patients [14]. Our study showed a prevalence of 14.6% for IDA which is similar to the number obtained from previous studies. This stresses that PHCs have a great role in diagnosing IDA and β-thalassemia. Additionally, it indicates that IDA and β-thalassemia contribute greatly to the burden of disease in the region.

Limitations

The main limitation of the study was attrition. Many patients presented for the first year of life screening but failed to follow up after the abnormal results were obtained. Another limitation was the decline in the amount of data available from PHCs during the COVID-19 pandemic period due to the nationwide quarantine laws issued by the government, leading to decreased attendance of parents at PHCs. The lack of inclusivity was another limitation, as the 13 PHCs did not have a representative sample of UAE nationals; on the contrary, they provided a proportionate representation of expats living in Dubai. The expat population may still be underrepresented since they may opt to screen their children at private clinics, as that may be more convenient.

## Conclusions

In conclusion, this study underlines the high prevalence of β-thalassemia and IDA in infants in the UAE, emphasizing the need for effective and reliable screening techniques. As shown in the study, the Mentzer index could be implemented as a screening tool for IDA and β-thalassemia as it showed promising sensitivity, especially when it comes to β-thalassemia screening; however, this study lacks the sample size necessary to produce clinically significant results. In the future, it is recommended to repeat the study on a larger, nationwide scale.
